# Challenges in Expanding Access to Dialysis in South Africa—Expensive Modalities, Cost Constraints and Human Rights

**DOI:** 10.3390/healthcare5030038

**Published:** 2017-07-31

**Authors:** Harriet Etheredge, June Fabian

**Affiliations:** 1 Wits Donald Gordon Medical Centre, 27 Eton Road, Parktown, 2193 Johannesburg, South Africa; june.fabian@mweb.co.za; 2 Department of Internal Medicine, Faculty of Health Sciences, University of the Witwatersrand, 7 York Road, Parktown, 2193 Johannesburg, South Africa

**Keywords:** dialysis, rationing, ethics, macroeconomics, human rights, resource constraints, South Africa

## Abstract

South Africa is a country with two distinct health sectors, which are both characterised by inequalities. Within this context, patients with end stage renal disease face unique and sometimes impenetrable barriers to accessing dialysis. There are a number of reasons for this situation. These include: the South African government’s endorsement of discordant, unequal policies, which disadvantage the most vulnerable; a lack of robust national guidelines; and divisive rationing practices, which are ad hoc and place the burden of responsibility for rationing dialysis on the clinician. In this paper, we trace the socio-economic mechanisms of how we have come to be in this situation, and overlay this with a detailed examination of South African legislation. Finally, we make comprehensive practical recommendations for rectifying the situation, which include engagement with key stakeholders, public–private partnerships, and more equitable funding mechanisms.

## 1. Definitions

Chronic kidney disease (CKD) is diagnosed using internationally accepted criteria, and defined as an estimated glomerular filtration rate <60 mL/min/1.73 m^2^ and/or persistent albuminuria [1]. There are five defined stages of kidney disease; the definition of CKD includes stages three to five. From stage three, it can be inferred that approximately 50% of kidney function may have been lost, and from stage three onwards, there is a significantly increased risk of morbidity and premature death [2].

End stage renal disease (ESRD) is the most severe form of CKD, and is also known as kidney failure. This is because at this stage, death is an inevitable consequence unless renal replacement therapy is instituted.

Renal Replacement Therapy (RRT) is comprised of treatment to replace, in part, the kidney function that has been lost so that life can be sustained. This is done with either dialysis therapy or a kidney transplant. For the purposes of this article, we are going to focus on dialysis. Transplantation and dialysis go hand-in-hand, so we will occasionally mention transplant in this article. However, we will not discuss transplant in any substantive detail, as it is beyond the scope of this paper to do so.

South African Rand (ZAR) is the South African unit of currency, which is known as the ‘Rand’ and internationally expressed with the abbreviation ZAR.

## 2. Introduction

### 2.1. CKD in Sub-Saharan Africa

The estimated prevalence of CKD in sub-Saharan Africa (SSA) is 13.9%, which is similar to global estimates of 13.4% [3,4]. However, incident CKD is predicted to rise disproportionately in SSA, where health transitions are characterised by rapid urbanisation, improved life expectancy, and population ageing. In this setting, both infectious and non-communicable diseases contribute risk. Poor infrastructure and the absence of screening and prevention programmes for kidney disease are systemic factors that further accentuate this risk [5,6,7]. As a result, CKD is often diagnosed at an advanced stage, when renal replacement therapy is essential to maintain life. Recently, the plight of those who require dialysis in SSA has been highlighted [8,9,10]. For the region, it is predicted that only 1.5% of those with diabetes and hypertension who need RRT will receive treatment. Almost three quarters of adults who start dialysis subsequently die due to late presentation at health care facilities, poor quality dialysis, or cessation of dialysis based on its prohibitive cost [8,9]. To add further perspective, the Global Burden of Disease Study reported a doubling of CKD-related deaths worldwide between 1990–2010, and in SSA, the years of life lost from CKD in 2010 were among the highest in the world [11].

### 2.2. Kidney Disease and Access to Dialysis in South Africa—Context and History

In South Africa (SA), very little is known about the prevalence of CKD and rates of progression to ESRD, but there are data regarding the provision of RRT across a sample of national registries. In a recent registry report, the most common cause of ESRD in adults was glomerulonephritis, followed by hypertensive renal disease and diabetic nephropathy. This demonstrates the contribution of both infectious and non-communicable diseases to the development of this condition [12].

In contrast to other SSA countries, SA was the first to provide access to kidney transplant and dialysis, which began in the 1960s. Initially, these services were primarily restricted to “white only” hospitals in large urban areas under the apartheid government, thereby excluding the majority of the population [13]. Despite a peaceful transition to democracy in 1994, and the adoption of a progressive constitution with an impressive bill of rights, South Africans still have limited access to dialysis care in the state sector. Isolated reports reveal that in state facilities, at least half of those who present for care are turned away [14,15]. In response to the high demand for care, some state centres have developed criteria for rationing that have been criticised by the broader nephrology community. These criteria are based on national citizenship, literacy, and issues linked to poverty, such as having insufficient funds to travel to a treatment centre. Poor adherence to medical regimens and apparently unfavourable home circumstances have also been cited as justifications to withhold access to care [14,15]. It could be argued that such rationing policies constitute a human rights violation and the clinicians ascribing to them are complicit, further compromising the most vulnerable in society.

This raises important issues for health policy. How do we equitably integrate dialysis, a highly specialised, resource-intensive treatment into the current health care system? Is this appropriate for SA? If so, how do we justify its prioritisation against numerous competing health care needs? The system in which the ill seek care must be transparent and accountable within a framework of progressive realisation, as provided by the South African Constitution [16].

The focus of this paper will be on the mechanisms currently in place to ration access to dialysis in SA. We argue that provision of care remains inadequate, and the progressive realisation of access to healthcare resources has not been achieved for those with ESRD.

## 3. Mechanisms of Rationing Access to Healthcare

Across the world, the provision of health care is expensive, and countries face challenges about how to most efficiently deliver a package of health services. Whether an upper-income country or a low-income country, governments are obliged to consider both the health needs of their populations, and the fundamental premises of their national health policies, and provide health services and interventions on this basis. This requires rationing, which necessitates the prioritisation of certain conditions over others, and perhaps the prioritisation of preventative over curative care. In relation to CKD, for example, the per capita gross domestic product (GDP) in the United States is three times greater than that of SA, yet both countries will still need to ration health care. As the United States is the only country in the world where dialysis is a constitutional right, the package of health care available to Americans may be dialysis-intensive compared to the healthcare package in SA. Rationing healthcare services is inevitable, but the manner in which this rationing takes place, e.g., the factors that are taken into account and the transparency of rationing policies, is the core issue. These factors can render rationing decisions ethical or otherwise.

## 4. Rationing Access to Dialysis in the South African Health System

The SA health system is distinctly two-tiered, with the private sector serving a smaller percentage (28–38%) of the population than the state sector (62–72%). Annual expenditure across sectors is almost equal, with ZAR 120.8 billion in the private sector, as compared to ZAR 122 billion in the state sector [17,18]. However, because of the higher patient burden, the state sector is considered under-resourced in all areas, including the provision of drugs, the availability of facilities, human resources, and equipment shortages [16]. Furthermore, the state health sector is considered poorly managed, and compromised by wasteful expenditure and corruption [19]. Those who access ‘fee-for-service’ health care in the private sector in SA enjoy an exponentially larger proportion of options than do those accessing government-funded care in the state sector [12]. Because private medicine is only available to those who are able to pay for it, wealthier, employed people are advantaged over the poorest and most vulnerable in society.

This manifestation of advantage can be clearly seen in the mechanisms that dictate the dialysis package available to state patients as compared to that available to private patients. In fact, the dialysis package of care provided to state patients is so substantively different from that provided to private patients, it is almost antithetical.

In the state sector, national guidelines published in 2009 mandate that only individuals who are eligible to receive a kidney transplant are accepted onto dialysis programmes, and that this is the primary mechanism for rationing [20]. The government’s justification for this policy is that there are limited national resources to fund dialysis in the state sector. This rationale was clearly mandated in the judgment of Soobramaney vs. the Minister of Health [21], a precedent-setting case regarding the right to access health services in a resource-constrained setting. Briefly, Mr Soobramoney presented with ischemic heart disease and cerebrovascular disease. He was in renal failure and had been receiving dialysis in the private sector. However, he exhausted his funding and applied for dialysis in the state sector. He was turned down on the basis that he was not eligible for a kidney transplant. The court found that the rationing of dialysis in this case was appropriate, given anticipated patient survival and resource scarcity.

In the well-resourced private sector, on the other hand, dialysis is considered a ‘prescribed minimum benefit’ (PMB) [22]. Prescribed minimum benefits relate to a statutory, pre-determined list of health conditions that private health funders are obliged to cover, regardless of the financial contributions made by the beneficiary. This means that private health providers may not refuse dialysis services to those who are able to pay for them (whether or not the insured person is eligible for a kidney transplant). Notably, this differential care across sectors is endorsed by the same national guidelines that restrict dialysis only to those eligible for transplant in the state sector [20]. The result of this policy is that there are significantly more people with ESRD who are defined as eligible for dialysis in the private sector, and this has driven the rapid expansion of private facilities. Had Soobramoney challenged a private healthcare funder, the judgment may very well have been in his favour.

**Figure 1:**
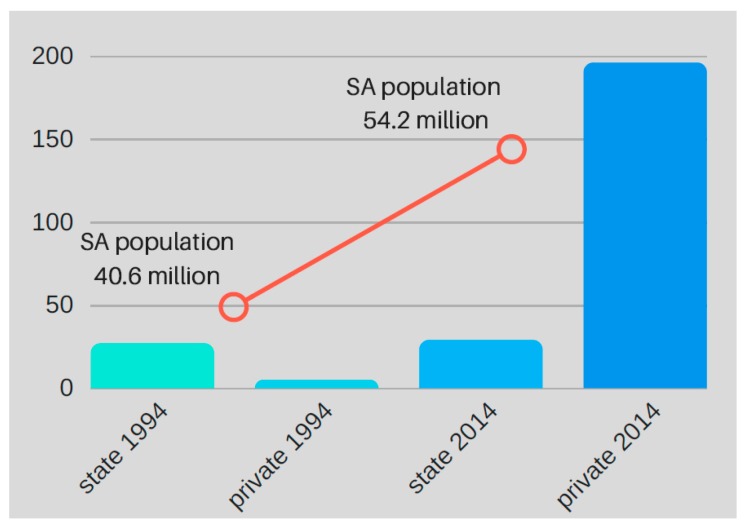
Comparative growth in state and private dialysis facilities in South Africa (1994–2014).

**Figure 2:**
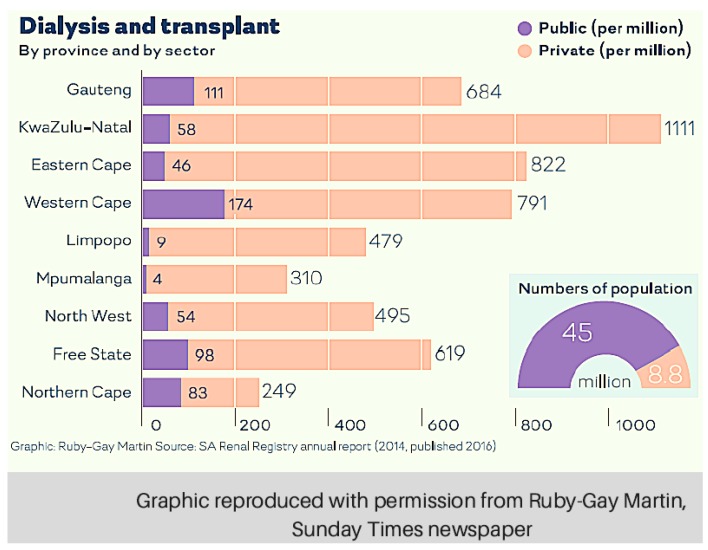
National treatment rates for ESRD in state and private sectors.

Taken a step further, the state can (indiscriminately) ration even those who are eligible for transplantation, based upon the poorly-defined rationale of ‘scarce resources’ [20]. We question upon what basis state and private sector policies were constructed, and how the government can justify the endorsement of policies that directly manifest in the iniquitous provision of health care. The outcome of these disparate policies across the two health sectors is evident when considering SA’s dialysis service statistics, based upon data provided by the South African Renal Registry. In 1994, SA’s first year as a democracy, there were markedly more dialysis facilities in state than private ones. For the 20-year period from 1994–2014, the overall number of state dialysis facilities has contracted (an absence of population-appropriate growth), whereas the number of private facilities has increased dramatically (by 3820%) (Figure 1 and Figure 2). The rapid growth of private sector dialysis facilities mirrors population growth, while the stagnation of facilities in the state sector does not correspond with population growth trends.

Figure 1 and Figure 2 depict disparities in access to RRT across sectors, and countrywide, since 1994 [12,23,24,25,26,27,28,29,30]. For a much more detailed analysis, please see the Table S1 in the Supplementary Materials.

Although it appears from Figure 1 that the growth of RRT facilities in the private sector has exceeded the population accessing them, in actual fact, the increase in dialysis volumes in the private sector in SA correspond with treatment rates achieved in other middle-income countries with similar GDPs. Table 1 illustrates the number of patients who are receiving chronic dialysis in countries with a similar GDP to SA. With 648 per million population (PMP) receiving dialysis in the private sector, management is similar to countries such as Bosnia-Herzegovina, which has a smaller GDP than SA, but a dialysis rate of 691 PMP. Similarly, Serbia, with a slightly larger GDP than SA, boasts a dialysis rate of 718 PMP. In context, the sluggish growth of dialysis in the state sector is much more worrying. The rate of 71 PMP is much lower than that seen in less wealthy countries such as Bangladesh, where a rate of 115 PMP has been achieved in spite of a GDP almost four times smaller than that of SA. A similar inference can be made for the Philippines. Hence, it is evident that although the observed growth in the private sector may seem excessive, the increase appears to be appropriately aligned with international efforts to increase access to dialysis.

## 5. How Has This Situation Come about?

There are a number of factors that, when viewed in conjunction with each other, may explain how discrepancies across the two health sectors have come about. These include legislation and its role in creating demand vs. supply side economic considerations, and the social determinants of health.

### 5.1. The Role of Legislation and Microeconomic Policy

The SA Constitution includes a bill of rights, which sets out a fundamental package of human rights that should ideally be enjoyed by all living in SA. Many of these rights are directly linked to resources and the provision of services. In terms of health care, the Constitution states that all people have the right to access health services. This should be read in conjunction with other rights, such as the right to life, the right to bodily and psychological integrity, and the right to freedom and security of the person. The Constitution also outlines the responsibility of the national government in promoting the realisation of human rights, with Section 27.2 of the Bill of Rights mandating that “The state must take reasonable legislative and other measures, *within its available resources, to achieve the progressive realisation* of each of these rights” [our italics] [32].

**Figure 3:**
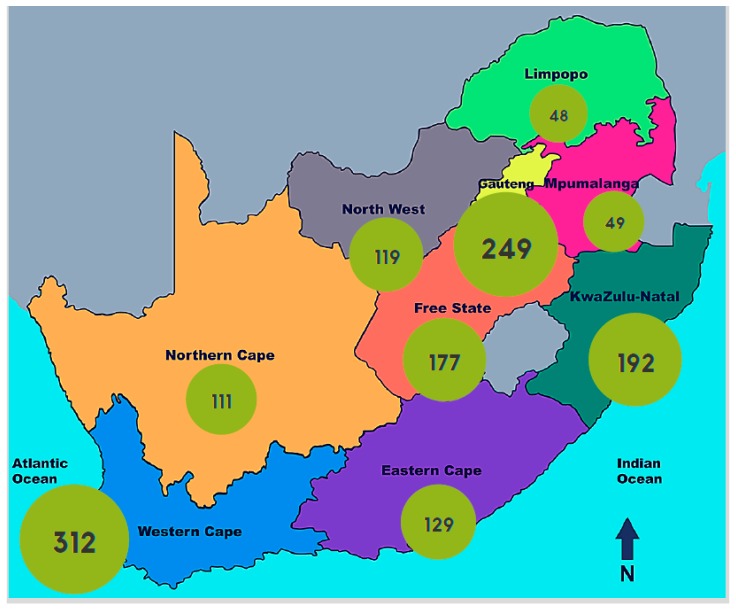
Geographical inequities in access to dialysis per million population in South Africa.

The notion of the progressive realisation of rights within available resources has some implications for the provision of healthcare services such as dialysis. Firstly, it is notable that the highest law of SA actually endorses rationing. By incorporating the notion of resource constraints, there is a tacit acknowledgement that every conceivable health service cannot be provided to every person. This is reasonable, and we are not taking issue with the concept of rationing in this article. Secondly, the Constitution mandates a *progressive* realisation of human rights, which suggests that the opportunities for those living in SA to enjoy their basic rights should grow over time. It is unfortunate that, when it comes to dialysis, the realisation of these rights in the state sector is vastly different to that in the private sector, as Figure 1, Figure 2 and Figure 3 demonstrate.

The apparent failure to implement access to RRT in a progressive fashion, as per the requirements of the Constitution, is magnified at the operational level in state hospitals, where there is an absence of guidelines to direct practitioners in the practical aspects of rationing. It is true that guidelines have been written for the Western Cape Province [33], but in those provinces where there are no guidelines for rationing, individual hospitals are compelled to devise their own policies, which are often inconsistent between sites. Given that these policies are not nationally or provincially endorsed, buy-in varies, and rationing decisions may ultimately depend largely on the clinician who is on duty when a patient presents to a unit. 

The private sector is different to the state sector, and operates within the strict, transparent legal framework of the Medical Schemes Act (No. 31 of 1998). The act is implemented and overseen by a vocal and active Medical Schemes Council, which is responsible for setting the legal minimum package of care that must be provided to all paying members of medical schemes. Since dialysis is considered a PMB, rationing access to it in the private sector is effectively non-existent. 

The lack of legislation in the state sector, especially compared to the rigorous regulation in the private sector, manifests in different patterns of health spending across the two sectors. The private sector is effectively demand-side driven; legally, the demand for RRT must always be met because of the PMB package. This accounts for the huge increase in the volume of private dialysis services since 1994 (Figure 1), and for the geographical diversity of these services (Figure 3). The state sector, on the other hand, is supply-side driven. Since there is no legislation obliging the government to fund dialysis, the supply of such services in the state sector is dismal. Ironically, this is not necessarily unconstitutional, because rationing within resource constraints is permitted. However, it would be hard to argue that there has been “progressive realisation” of the right to access dialysis in the state sector. Progressive realisation would, at bare minimum, accommodate a population-appropriate growth in facilities and services. Sadly, since 1994, dialysis services have only grown by 7.7%, while the population obliged to access care in the state sector has increased by 38% (32.98 million in 1994–45.2 million in 2014) [30]. Thus, it appears that the regressive realisation of the right to access dialysis services is taking place in the state sector. This is unconstitutional.

### 5.2. The Role of the Social Determinants of Health

Socio-economic and socio-demographic factors seem to be significant determinants of the extent to which dialysis services can be accessed, and by whom. The notion of Social Determinants of Health may also explain how inequalities in access have come about [14]. Broadly, the following are identified as social determinants of health [34]: income, education, occupation, social class, gender, ethnicity and socio-political context. The World Health Organisation (WHO) presents several pathways for the social determinants of health, and notes that differences in health are seen most acutely in settings where there are disparities in power relations and socio-economic status [34]. SA exemplifies these circumstances, particularly when one considers the resource differential between the state and private sectors. It is argued that those of lower socio-economic status are not always able to make life decisions that are consistent with maintaining health. Hence, lower socio-economic groups are perceived to be less healthy [34]. There seems little doubt that the current status quo for accessing dialysis in SA disadvantages the poor, as previous SA research has shown [14].

The role of income, education and occupation in determining whether an individual in SA will be able to access dialysis is clearly seen in factors affecting the membership of private medical schemes. Generally, formal employment is a pre-requisite for accessing medical scheme cover, because it affords an individual the opportunity to join a medical scheme independently, or membership of a medical scheme is included in an employment package. Because access to dialysis is a PMB, it is more likely that those who are formally employed will be able to access dialysis in the private sector. However, private medical schemes also limit the extent to which they fund PMBs. Hence, out-of-pocket expenses may also be incurred by medical scheme members, and the lowest-earning members will then be disadvantaged. Furthermore, SA boasts a large informal employment sector, where individuals are not necessarily afforded the opportunity to join a medical aid scheme because, although employed, they do not earn enough money to cover an expensive medical aid membership.

The role of socio-political context in determining access to dialysis can be seen when considering the geographical location of advanced health facilities (Figure 3). Those with the infrastructure and resources to provide dialysis are overwhelmingly clustered in large urban areas such as SA’s main cities [35]. These urban areas have historically been—and continue to be—much wealthier than the rural areas of SA, resulting in easier access to dialysis for urban-dwelling individuals (whether state or privately funded). Furthermore, legislation that was historically inequitable under the Apartheid system may also still be manifest in patterns of access to healthcare services and the location of such services, clustered in urban areas populated primarily by individuals of white ethnicity during Apartheid. For less-wealthy individuals living in poorer, rural areas, these services are not readily available. Furthermore, accessing care at an urban facility may be prohibitively expensive for those from rural areas due to the regular transport requirement and the significant distance that patients need to travel. Here again, those who are worst off are further disadvantaged by systemic geographical barriers from accessing equitable health care [35].

There are other factors that result in asymmetries in access to dialysis for the SA population. However, it is very clear that structural issues regarding the health system, persistent historical inequities in the location of health facilities, and legislative discrepancies have resulted in inconsistent practices, where dialysis in SA is severely rationed for the poor, and in excellent supply for the wealthy.

## 6. Ethical Implications

A major ethical challenge for any government, especially in a constitutional democracy such as SA, is how to facilitate a health system that is fair and upholds the principles of justice. Justice is as much an ideal of an equitable society as it is ‘in the eye of the beholder’. As such, justice also involves accountability and, when individuals across society perceive that they are not being treated fairly, it is the responsibility of policymakers to account for it. 

Hence, ethical and just health systems rely not only on fair policies, but also on the dissemination of accurate and unambiguous information regarding these policies to patients. This promotes both autonomy and patient centeredness, because as patients engage with the information provided, there is an opportunity to manage expectations. This is particularly important in all systems where rationing is inevitable. In the absence of national legislation or robust guidelines detailing the processes by which access to dialysis is rationed, state patients are unable to engage with the system, and will never be able to say for certain where they stand in relation to gaining access to dialysis should they need it. This introduces a problematic element of uncertainty, which is accompanied by feelings of desperation, indignation and being treated ‘unfairly’. Uniform guidelines for rationing access to dialysis across SA would mitigate some of these factors, as patients would be able to ascertain their likelihood of accessing treatment one way or another, and allegations of unfairness may be mitigated if all patients are selected based on the same set of guidelines. Having robust, nationally applicable guidelines also gives patients some recourse to legal challenge when they feel that access to healthcare resources has been unfair.

Moreover, ethical health systems have obligations not only to patients, but also to the healthcare professionals who work within them. Amongst others, there is a mandate to be able to work in a safe environment, and practice within the scope of one’s training. One of the unfortunate consequences of a lack of uniform rationing policy for dialysis in SA is that rationing decisions are often made by individual clinicians, or sometimes a small team. In the absence of robust guidelines, patients may perceive that clinicians are independently responsible for whether or not they received dialysis, rather than the government, who is ultimately responsible for providing it.

## 7. Recommendations—A Comprehensive National Strategy for Kidney Disease

**Figure 4:**
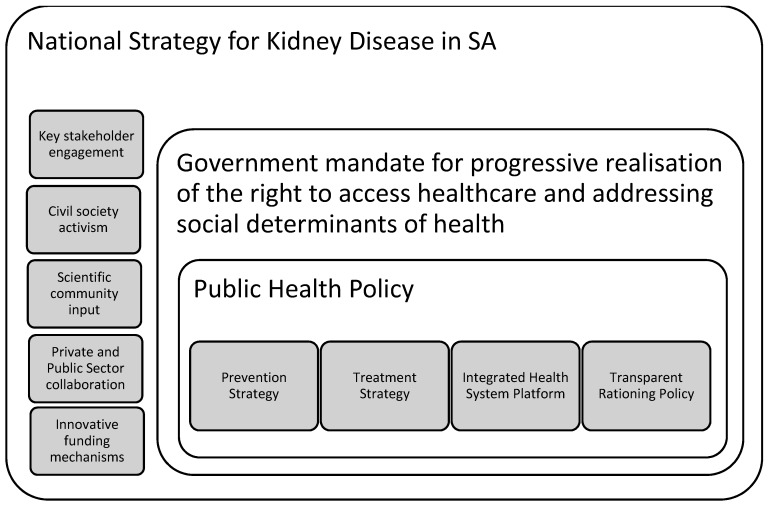
National strategy for kidney disease.

A comprehensive, inclusive national strategy for managing CKD in SA may be a good framework within which stakeholders can engage, guidelines can be drawn up and ratified, and collaborations can be formalised. A suggested conceptual framework for a comprehensive national strategy is depicted in Figure 4. This framework accounts for the mandate of progressive realisation, and proposes a number of aspects that could comprise a robust public health approach to facilitating access to dialysis and the management of kidney disease. 

A national strategy for the management of CKD needs to account for challenges both at macro level and at the clinic level. In order to address these challenges, a number of groups need to engage both at the national and public health policy levels. These stakeholders include civil society, the scientific community, the government, and the private health sector. Engagement would need to address systemic challenges in providing CKD management, such as facilitating the progressive realisation of the right to access health care, and addressing the social determinants of health. At a policy level, prevention and treatment strategies should go hand-in-hand, implemented in an integrated health system, and guided by a transparent rationing policy. All decisions and discussions would need to critically explore the scope for instituting novel funding strategies to expand access.

### 7.1. Key Stakeholder Engagement

In order to formulate a national policy that has the support of all key role-players in the management of CKD in SA, it will be necessary to invite all parties to collaborate towards a common goal. These parties should include the private sector, state sector, the Department of Health, medical aid schemes, the South African Dialysis Association, human resources specialists, technologists, nephrologists, dialysis nurses, ethicists, legal experts, and community representatives. Robust engagement along these lines could help to formulate platforms for both the prevention and treatment of CKD, and could facilitate public–private partnerships, which may be essential in expanding the services that are available. 

### 7.2. Civil Society Activism

The example of access to antiretroviral medication to prevent mother-to-child transmission of HIV highlights the need for civil society activism and the power of social mobilisation. The ground-breaking case of the Treatment Action Campaign vs. The Minister of Health was heard at Constitutional Court level, where the human rights principles that have been discussed in this paper are tested [36]. Clearly, some of the challenges in accessing dialysis are rights-based, and similar action brought by the renal community and civil society activists may have the benefit of not only increasing access to care, but also clarifying the responsibilities of government in the progressive realisation of resources.

### 7.3. Scientific Community Input

In order to justify national strategies, input from the scientific community regarding the burden of CKD and optimal modes of management will be required. At present, there is very little scientific research into CKD at a population level in SA. It would be helpful if grant funding was made available to facilitate the collection of this type of data. The South African Renal Registry, which is published annually, is certainly a reliable barometer of the situation. However, greater scientific involvement is required to formulate evidence-based solutions.

### 7.4. Private–Public Partnerships (PPP)

Countries with very similar health systems to SA have made remarkable progress in improving access to chronic dialysis in spite of rationing through public–private partnerships (PPPs).

The example of Malaysia is enlightening. Malaysia is comparable to SA, with a government–funded health sector and a private sector funded through much the same means as the SA private sector. By introducing PPPs, the number of Malaysian patients on dialysis grew from 46 PMP in 1990 to 512 PMP in 2005. A number of different collaborative financing mechanisms, which received substantial governmental support, were instrumental in bringing about this change. 

It is interesting to note that as of 2013, the GDP of Malaysia was slightly less than SA, with USD 313.2 billion in Malaysia vs. USD 350.6 billion in SA [37]. Although there are some differences in population number and CKD profiles between the two countries, this example from Malaysia clearly illustrates how committed, dedicated government buy-in can have a positive effect. Thus far, there is little willingness from the SA government to actually engage on this issue, despite committing to the pursuit of such arrangements in a national guidelines document in 2009 [20].

Public–private partnerships could also assist in overcoming some of the most insidious social determinants of health. For instance, it could facilitate access to dialysis in areas where there is a private facility but no state facility. This would decrease the travel time and expense for state patients without necessitating that government build an entirely new facility. At a summit for the management of CKD in SA, the government did affirm its commitment to this type of collaboration.

### 7.5. Public Health Policy

Ideally, a comprehensive approach to CKD in SA would include collecting and disseminating reliable information on the burden of this condition, which would accurately inform policymakers in formulating a national strategy. A national strategy should include public education, screening programmes for those at risk of CKD to facilitate effective primary and secondary interventions, and the provision of access to RRT for those who develop ESRD. To achieve this, accurate information is required from the scientific community, national guidelines must be applicable across both sectors, and a combination of cost-effective prevention and treatment strategies need to be prioritised. Both primary and secondary prevention will have to be provided within an integrated health care framework for infectious and non-communicable conditions. Increasing the capacity for RRT requires expansion of dialysis and transplantation services in tandem, as success is defined by access to both.

At present, the predominant focus of care (by clinicians and funders) is dialysis therapy. This is problematic for a few reasons, the most important being that irrespective of resources, the cost of dialysis precludes most who need it. In SA, kidney transplant rates remain very low, so that patients are subjected to much longer periods of dialysis while awaiting transplant. Aside from the financial cost, this substantially compromises their health. In the state system, low transplant rates also prevent others from accessing care as the number of dialysis slots is fixed, which worsens the inequity.

## 8. Conclusions

The current predicament in the provision of dialysis services for ESRD, within the broader framework of care for CKD in SA, as explored in this paper, goes beyond service delivery and policy. The government’s failure to progressively realise access to dialysis services for the majority of South Africans is a human rights issue that is worthy of legal challenge at the Constitutional level. It would be prudent for the government to commit to addressing the regression of dialysis services in the state sector as a first step towards building a national framework for the management of CKD in SA. 

In the absence of such a commitment, we are left with a lingering question: “Can we say that we are doing the best we can to manage CKD in SA?” We think the answer is “No”.

## Figures and Tables

**Table 1:** Prevalence of chronic dialysis across some upper–middle income countries.

Country	Gross Domestic Product ^1^ (Int$)	Chronic Dialysis/(pmp) ^2^	World Bank Income-Level Classification
Bangladesh	2942	115	Low income
Philippines	6587	221	Lower–middle income
Bosnia-Herzegovina	10,202	691	Upper–middle income
Thailand	15,435	998	Upper–middle income
Columbia	12,725	487	Upper–middle income
South Africa	12,859	719 ^3^	Upper–middle income
Brazil	15,814	557	Upper–middle income
Serbia	13,772	718	Upper–middle income

^1^ GDP and income classification from The World Bank: http://data.worldbank.org/indicator/NY.GDP.PCAP.CD. ^2^ Prevalence rate of ESRD in 2013 as measured by number of patients receiving chronic dialysis/per million population (pmp) [31]. ^3^ 71 pmp for the state sector and 648 pmp for the private sector [24].

## Supplementary Materials

The following are available online at www.mdpi.com/2227-9032/5/3/38/s1.
