# Supplementary material for: Challenges in Expanding Access to Dialysis in South Africa—Expensive Modalities, Cost Constraints and Human Rights
Source: Healthcare (Basel). 2017 Jul 31;5(3):38. doi: 10.3390/healthcare5030038 (PMC5618166; doi:10.3390/healthcare5030038)
Supplement: Supplementary file 1 [file healthcare-05-00038-s001.docx]

**Table S1.** Availability of dialysis facilities across sectors and provinces of South Africa.

| 1994 | | | 2012 | | | 2013 | | | 2014 | | |
| --- | --- | --- | --- | --- | --- | --- | --- | --- | --- | --- | --- |
|  | S | P |  | S | P |  | S | P |  | S | P |
| Transvaal | 11 | 4 | Gauteng | 6 | 50 | Gauteng | 6 | 52 | Gauteng | 6 | 63 |
| Cape Province | 10 | 0 | Eastern Cape, Northern Cape and Western Cape | 9 | 42 | Eastern Cape, Northern Cape and Western Cape | 9 | 45 | Eastern Cape, Northern Cape and Western Cape | 9 | 45 |
|  |  |  |  |  |  |  |  |  |  |  |  |
| Natal | 3 | 1 | KwaZulu-Natal | 4 | 43 | KwaZulu-Natal | 5 | 49 | KwaZulu-Natal | 5 | 50 |
| Freestate | 3 | 0 | Freestate | 6 | 9 | Freestate | 6 | 9 | Freestate | 6 | 11 |
|  |  |  | Limpopo | 0 | 6 | Limpopo | 0 | 7 | Limpopo | 0 | 7 |
|  |  |  | Mpumalanga | 0 | 6 | Mpumalanga | 0 | 7 | Mpumalanga | 0 | 9 |
|  |  |  | North West | 3 | 7 | North West | 3 | 8 | North West | 3 | 11 |
| Total | 27 | 5 | Total | 28 | 163 | Total | 29 | 178 | Total | 29 | 196 |
